# A Case of Nebulin-Related Nemaline Myopathy With Asymmetric Distal Lower Limb Weakness

**DOI:** 10.7759/cureus.78945

**Published:** 2025-02-13

**Authors:** Hironori Mizutani, Yohei Misumi, Kohei Hamanaka, Nozomu Tawara, Satoko Miyatake, Naomichi Matsumoto, Mitsuharu Ueda

**Affiliations:** 1 Department of Neurology, Graduate School of Medical Sciences, Kumamoto University, Kumamoto, JPN; 2 Department of Human Genetics, Yokohama City University Graduate School of Medicine, Yokohama, JPN; 3 Department of Human Genetics, Yokohama City University, Yokohama, JPN

**Keywords:** asymmetric muscle weakness, distal myopathy, nebulin gene, nemaline myopathy, whole-exome sequencing

## Abstract

We report the case of a 37-year-old female who presented with asymmetric, distal muscle weakness in the lower limbs, which had its onset in childhood. Muscle biopsy revealed pathological changes consistent with nemaline myopathy, and suspected biallelic variants in the *nebulin* (*NEB*) gene, NM_001271208.1:c.24684G>C p.(Ser8228Ser) and c.23847+164A>G were identified. *NEB*-related myopathy typically presents with symmetric, proximal-dominant muscle weakness and atrophy. However, reports of nemaline myopathy with distal-dominant muscle involvement are rare. This case exhibited a marked asymmetric, distal-dominant myopathy in the early stages of the disease, and it may contribute to our understanding of the genotype-phenotype correlation of pathogenic *NEB* variants.

## Introduction

Nemaline myopathy was first described by Shy et al. in 1963 [1]. Nemaline bodies (also referred to as rods) appear red under Gomori-trichrome staining in light microscopy and as dark, lattice-like structures under electron microscopy [1]. Nemaline myopathy is associated with numerous causative genes, including *nebulin (NEB)*, *ACTA1*, *CFL2*, *TPM2*, and *LMOD3*. Among these, *NEB*-related myopathy is the most common cause of nemaline myopathy, accounting for approximately 50% of cases [2]. Its onset can range from the neonatal period to adulthood, with a wide spectrum of clinical manifestations varying from mild to severe. Typically, it presents with symmetric, proximal-dominant muscle weakness. We report a case of nemaline myopathy with marked asymmetry and predominantly distal lower limb muscle weakness caused by suspected biallelic variants in the *NEB* gene.

## Case presentation

A 37-year-old female with gait disturbance was referred to our hospital. The patient had no significant medical or family history of similar symptoms, and her parents were non-consanguineous. Her birth and developmental milestones had been normal. She had experienced difficulty with running at the age of three, begun tripping with her left foot at age six, and developed a noticeable left foot drop by age eight. In her 20s, she had begun tripping with her right foot and experienced fatigue in her neck and arms. At around age 23, she had started tripping with her right foot as well. These symptoms had gradually worsened, leading to a suspicion of distal myopathy, and she was hospitalized at age 37.

On examination, she was alert, with no abnormalities in higher brain function. Facial muscle strength was normal, and no articulation or swallowing difficulties were noted. Manual muscle testing (MMT) revealed a score of 4/5 for the right anterior tibial muscle and 0/5 for the left, indicating marked asymmetry in muscle weakness and atrophy. Additionally, left pes equinus and cavus were noted. The strength of the gastrocnemius and soleus muscles was preserved. Mild muscle weakness, with an MMT score of 4/5, and mild atrophy were observed symmetrically in the sternocleidomastoid, neck flexors, deltoid, and iliopsoas muscles. Deep tendon reflexes were diminished in the upper limbs and absent in the lower limbs, with no pathological reflexes. She was able to walk without a cane, and her gait was characterized by a left-sided steppage gait.

**Figure 1:**
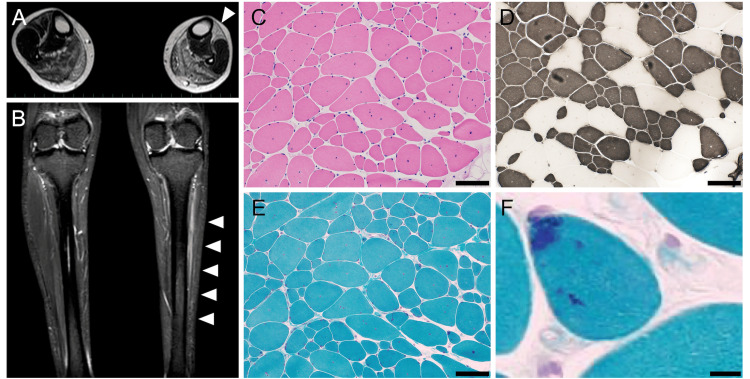
MRI images and muscle histology Skeletal muscle MRI demonstrated muscle atrophy and fatty replacement of the lower extremity muscles. Notably, asymmetric atrophy in the anterior tibial muscle was observed (arrowheads) (A, B). Representative pathological findings from the right deltoid muscle: marked variation in fiber size on hematoxylin and eosin staining (C), type 1 fiber atrophy and predominance on ATPase staining at pH 4.2 (D), and scattered fibers containing nemaline bodies on modified Gomori-trichrome staining (E, F). Scale bars: 100 μm (C-E) and 10 μm (F) MRI: magnetic resonance imaging

Blood tests showed normal creatine kinase levels (82 IU/L, normal range: <153 IU/L). Cardiac function tests were normal, but pulmonary function testing revealed a reduced vital capacity (%VC, 68.6%) without any symptoms. Nerve conduction studies showed markedly reduced compound muscle action potential amplitudes in the peroneal nerve. Needle electromyography showed significantly reduced amplitudes in the tibialis anterior. MRI of the skeletal muscles revealed pronounced asymmetry in muscle atrophy in both lower legs, with high signal intensity on T2-weighted imaging and short tau inversion recovery sequences (Figures 1A, 1B).

Muscle biopsy samples were obtained from the right deltoid muscles (Figures 1C-1F). Hematoxylin and eosin staining revealed variation in muscle fiber size without evidence of necrosis or regeneration, and numerous muscle fibers contained internal nuclei. Gomori-trichrome staining identified abundant nemaline rods, a hallmark histopathological feature of nemaline myopathy. Whole-exome sequencing revealed suspected biallelic *NEB* variants, NM_001271208.1:c.24684G>C p.(Ser8228Ser) and c.23847+164A>G. Although the former was of maternal origin, the latter could not phase these variants because of the absence of the paternal DNA. No other pathogenic variants associated with hereditary myopathy were detected.

RNA sequencing analysis demonstrated that c.24684G>C caused increased retention of intron 175, while c.23847+164A>G created a novel splicing donor site, and these variants were shown to be pathogenic by Hamanaka et al. [3]. According to the American College of Medical Genetics and Genomics/Association for Molecular Pathology (ACMG/AMP) 2015 guidelines, the variants were classified as likely pathogenic [4]. Based on the characteristic pathological findings and reference to previous reports, we diagnosed this case as nemaline myopathy, caused by suspected biallelic *NEB* variants. Since no specific treatment exists for this disease, symptomatic management and rehabilitation were provided. The symptoms progressed slowly over time. At a follow-up at the age of 45, the patient retained the ability to walk and maintained independence in daily activities.

## Discussion

The clinical severity of nemaline myopathy caused by pathogenic *NEB* variants varies widely, and this variability is closely associated with the amount and size of nebulin protein expressed in muscle tissue, as well as the retention of actin and tropomyosin binding sites [5]. *NEB*-related myopathy is typically caused by homozygous or compound heterozygous variants; however, in rare cases, dominant variants have also been reported [6]. Beyond histologically defined nemaline myopathy, pathogenic variants in *NEB* may lead to additional clinical manifestations, including core-rod myopathy, distal nebulin myopathy without nemaline rods, lethal multiple pterygium syndrome, and a dominant form of distal nemaline/cap myopathy [6,7]. The typical clinical presentation of *NEB*-related myopathy involves initial proximal muscle weakness, which may later progress to include distal muscle weakness [8].

Although less common, *NEB*-related myopathy with distal-dominant muscle weakness, as observed in this case, has been documented (Table 1) [7, 9-16]. The onset of symptoms most commonly occurs during childhood or young adulthood, with an average age of 16.7 years. Various homozygous, compound heterozygous, and heterozygous variants have been reported. In all these cases, patients retained the ability to walk, and the clinical symptoms were generally milder compared to typical *NEB*-related myopathy. Pathological findings in 16 out of 22 cases revealed the presence of characteristic nemaline bodies, with no cases demonstrating elevated creatine kinase levels. Consistent with our case, all reported cases exhibited severe impairment of the tibialis anterior muscle, although the gastrocnemius muscle remained relatively preserved in 18 of 22 cases [10]. A distinctive feature in our case was the marked asymmetry in muscle atrophy and weakness of the anterior tibial muscle. Interestingly, eight of 22 previous cases of *NEB*-related myopathy presenting with distal-dominant muscle weakness exhibited a notable asymmetric distribution in muscle weakness (Table 1).

**Table 1:** Clinical features of NEB-related myopathy with distal-dominant muscle weakness from previous reports GS: gastrocnemius; ND: no description; TA: tibialis anterior

Variant	Ethnicity	Sex	Age at evaluation (y)	Age at onset (y)	TA weakness	GC weakness	Asymmetry in muscle weakness	Nemaline bodies	Reference
ND	English	M	6	6	+	-	-	+	Scoto et al. [9]
c.24372_24376dup									
g.231441_231442del	French	F	61	6	+	-	-	+	Lehtokari et al. [10]
g.220502T>C									
g.43846dup	Hungarian	M	11	11	+	+	-	+	Lehtokari et al. [10]
g.47618G>A									
g.207181A>C, homozygous	Finnish	M 4, F 1	57, 48, 70, 42, 59	30, 10, 57, 6, childhood	5/5	0/5	-	1/5	Wallgren et al. [11]
g.171944G>T, homozygous	Finnish	M 1, F 1	37, 42	Infant, 30	2/2	0/2	-	1/2	Wallgren et al. [11]
g.163689-2A>G	French	M	17	Infant	+	-	-	+	Malfatti et al. [12]
c.24294_24297dup									
c.19944G > A	French	F	22	1	+	-	-	+	Malfatti et al. [12]
chr2.hg19:g.(152,465,598_152, 465, 794)									
c.24190_24193dup	French	M	14	Infant	+	-	-	+	Malfatti et al. [12]
c.11601+5G > A									
c.3387del	Korean	M	43	38	+	+	+	+	Park et al. [13]
c.24580G>A									
c.3387del	Korean	M	9	5	+	+	-	+	Park et al. [13]
c.4617-4629del									
c.20131C>T	Japanese	M	37	10	+	+	+	+	Mizuno et al. [14]
c.9046C>T									
c.23161A>T	Japanese	M	35	32	+	-	+	+	Mizuno et al. [14]
c.20132C>T									
c.20131C>T	Japanese	F	65	33	+	-	+	+	Ohara et al. [15]
c.674C>T									
chr2:g.(152454645_152456955)_(152554712_152561404)del	Finnish	M 1, F 2	30, 57, 73	3, 35, 10	3/3	0/3	+	2/3	Kiiski et al. [7]
g. = /(152427326_ 152427830)_(152567183_152567194)del	Finnish	F	26	4	+	-	+	-	Sagath et al. [16]
c.24684G>C	Japanese	F	37	8	+	-	+	+	Present case
c.23847+164A>G									

The cases reported by Sagath et al. and Kiiski et al. involved large heterozygous deletions in *NEB* exons, and they proposed that the dominant-negative effect of a smaller-than-normal nebulin protein could be the likely cause of nemaline myopathy in these patients [7]. Similarly, recent reports have documented rare instances of nemaline myopathy associated with *NEB* variants that present with significant asymmetric muscle atrophy and weakness, as in the current case. More recently, both dominantly inherited distal myopathy and a recessive congenital asymmetric distal myopathy with hemifacial weakness have been linked to large deletions in *NEB*, further expanding its clinical spectrum [7,16]. Mosaic deletions, which exhibit varying levels of mosaicism across tissues, could contribute to the observed asymmetry in muscle weakness [7]. Including our case, instances with a marked asymmetric distribution of muscle weakness, particularly those involving compound heterozygous or heterozygous variants, may support this hypothesis. These findings underscore the clinical significance of asymmetric, distal‐dominant muscle weakness as a rare phenotype in *NEB*‐related myopathy, potentially enabling earlier diagnosis and more precise clinical classification.

## Conclusions

We discussed a case of nemaline myopathy with suspected biallelic *NEB* variants characterized by pronounced asymmetric, distal-dominant muscle atrophy and weakness, underscoring the importance of recognizing this phenotype to enhance diagnostic accuracy. The distinctive distribution of muscle weakness and marked asymmetry observed in this case is crucial for elucidating the genotype-phenotype correlation of this condition.
